# The Role of Cargo Proteins in GGA Recruitment

**DOI:** 10.1111/j.1600-0854.2007.00556.x

**Published:** 2007-03-26

**Authors:** Jennifer Hirst, Matthew N J Seaman, Sonja I Buschow, Margaret S Robinson

**Affiliations:** 1 Cambridge Institute for Medical Research, University of Cambridge Cambridge CB2 0XY, UK; 2 Current address: Department of Biochemistry and Cell Biology, University of Utrecht Utrecht 3508 TD, The Netherlands

**Keywords:** AP-1, cation-independent mannose 6-phosphate receptor, clathrin, coated vesicle, TGN

## Abstract

Coat proteins are recruited onto membranes to form vesicles that transport cargo from one compartment to another, but the extent to which the cargo helps to recruit the coat proteins is still unclear. Here we have examined the role of cargo in the recruitment of Golgi-localized, γ-ear-containing, ADP ribosylation factor (ARF)-binding proteins (GGAs) onto membranes in HeLa cells. Moderate overexpression of CD8 chimeras with cytoplasmic tails containing DXXLL-sorting signals, which bind to GGAs, increased the localization of all three GGAs to perinuclear membranes, as observed by immunofluorescence. GGA2 was also expressed at approximately twofold higher levels in these cells because it was degraded more slowly. However, this difference only partially accounted for the increase in membrane localization because there was a approximately fivefold increase in GGA2 associated with crude membranes and a ∼12-fold increase in GGA2 associated with clathrin-coated vesicles (CCVs) in cells expressing CD8-DXXLL chimeras. The effect of cargo proteins on GGA recruitment was reconstituted *in vitro* using permeabilized control and CD8-DXXLL-expressing cells incubated with cytosol containing recombinant GGA2 constructs. Together, these results demonstrate that cargo proteins contribute to the recruitment of GGAs onto membranes and to the formation of GGA-positive CCVs.

The function of coat proteins is to sort cargo proteins into vesicles, which then transport the cargo from a donor to an acceptor membrane. However, there is some controversy over the role that cargo proteins play in their own sorting. Clearly, they interact with coat proteins on the donor membrane in order to get packaged into vesicles. But do the cargo proteins also help to get the coat proteins onto the membrane in the first place?

Over the years, there have been a number of studies addressing this issue. Initially, there was a tendency to assume that coat proteins were recruited onto membranes solely or primarily through their interactions with cargo. However, this view failed to take into account the observation that at steady state, cargo proteins are often much more abundant in the acceptor membrane than in the donor membrane (as would be expected if the coat proteins were doing their job efficiently), yet the coat proteins are only recruited onto the donor membrane. In addition, the discovery of other binding partners for coat proteins, such as small GTPases and phosphoinositides, which clearly play a role in their recruitment, has led to the idea that coat protein recruitment and cargo selection may be two independent events [reviewed by Robinson (1)].

At the other extreme, there is the view that the role of the cargo proteins is a completely passive one and that the coat proteins are recruited onto membranes to form vesicles whether or not there is any cargo for them to sort [e.g. see Santini et al. (2)]. In other words, the coated vesicle could be analogous to either an elevator or an escalator. If the coated vesicle were like an elevator, it would need passengers to trigger its formation. However, if it were like an escalator, it would form regardless of passengers, and the passengers would simply hop onto a conveyance that was already in operation.

Thus, whether the cargo plays an active or a passive role in its own sorting is still a matter of some debate, and there are experimental data to support both sides. Early studies reported that overexpression of the transferrin receptor, perhaps the best characterized of all the cargo proteins packaged into endocytic clathrin-coated vesicles (CCVs), leads to an increase in the amount of clathrin coating on the plasma membrane in both mouse L cells (3) and chick embryo fibroblasts (4). The transferrin receptor uses a YXXΦ motif to bind to the adaptor protein (AP)-2 complex, the major clathrin adaptor at the plasma membrane, and this suggested that the increased amounts of receptor might lead to increased recruitment of AP-2, which in turn would lead to increased recruitment of clathrin. However, in subsequent studies, no such effects were observed in HeLa cells, either when the transferrin receptor was overexpressed ∼20-fold (5) or when chimeric constructs with different cytoplasmic tails, including one with a YXXΦ motif, were overexpressed (2). Possibly, the differences are because of cell type, but the earlier studies were never revisited and the general consensus now seems to be that cargo proteins do not play a major role in coat protein recruitment at the plasma membrane. This does not rule out the possibility that the cargo proteins might help to stabilize the coat proteins once they have been recruited and, indeed, a recent live-cell imaging study, in which temporal as well as spatial events could be analyzed, suggested that cargo capture might play a key role in the commitment of a coated pit to go on to become a coated vesicle (6).

There have also been conflicting reports about the role of cargo in the recruitment of coats onto intracellular membranes. The major intracellular adaptor for CCVs is the AP-1 complex, which like the AP-2 complex binds YXXΦ motifs, and the best characterized of its cargo proteins are the cation-independent mannose 6-phosphate receptors (CIMPR) and cation-dependent mannose 6-phosphate receptors. Early studies reported that there was a reduction in the amount of AP-1 associated with membranes in cells from a double knockout mouse lacking both mannose 6-phosphate receptors (MPRs) and that overexpression of the CIMPR caused a modest increase in AP-1 recruitment (7,8). However, these data were called into question by a subsequent study, in which the authors concluded that the amount of AP-1 associated with membranes did not change in MPR-deficient cells, but that there were gross alterations in the morphology of the cells, which made it difficult to interpret the immunofluorescence images (9). The role of cargo proteins in the budding of a different kind of coated vesicle, the COPII vesicle, which transports newly synthesized proteins out of the endoplasmic reticulum, has also been examined, by treating cells with cyclohexamide to block *de novo* protein synthesis. Again, the treatment had no apparent effect on COPII vesicle budding, leading the authors to conclude that the vesicles continued to bud regardless of the amount of cargo available (10).

One coat component for which the role of cargo proteins in recruitment has not yet been examined is the GGA family of proteins (11–13). The Golgi-localized, γ-ear-containing, ADP ribosylation factor (ARF)-binding proteins (GGAs) act as monomeric adaptors primarily at the *trans* Golgi network (TGN), although they have also been localized to endosomes (14). There are three GGA genes in mammals and two in the budding yeast *Saccharomyces cerevisiae*, and there is now abundant evidence that the GGAs help to package cargo into CCVs in both organisms (12,^13^,^15^–22). However, so far they have not been detected in Western blots of purified CCVs (13), possibly because their association with membranes is extremely labile (19). The relationship between GGAs and AP-1 is also unclear: their localization patterns are similar but not identical, with the GGAs residing more in perinuclear/TGN membranes and AP-1 having a more peripheral distribution (23). Various ideas have been proposed to explain how the two might cooperate, including suggestions that they may operate on sequential pathways (e.g. GGAs might hand over certain cargo proteins to AP-1), on parallel pathways (e.g. to transport different cargo proteins between the same compartments) or on opposite pathways (e.g. GGAs might function mainly in the TGN to endosome direction and AP-1 in the reverse direction) (24).

The GGAs consist of three folded domains: a VHS domain, a GAT domain and a domain that is homologous to the C-terminal appendage or ‘ear’ domain of the γ-subunit of the AP-1 complex, sometimes called the GAE domain. The three folded domains are joined together by unstructured loops, the second of which is very long and contains clathrin-binding sites, similar to the flexible loops connecting the N- and C-terminal domains of the large subunits of the AP complexes. Binding partners have now been identified for all three of the folded domains [reviewed by Bonifacino (25)]. The VHS domain binds to cargo proteins with DXXLL motifs, such as the two MPRs and sortilin. The GAT domain binds to ARF, ubiquitin, rabaptin-5 and Tsg101. The C-terminal appendage domain binds to some of the same partners as the γ-appendage, although with the exception of p56, which colocalizes mainly with the GGAs, most of these proteins preferentially associate with AP-1 *in vivo*(23). The most important of the three GGA domains for determining localization appears to be the GAT domain. Purified recombinant full-length GAT domain or a 46-residue fragment containing the ARF-binding site can be recruited onto membranes *in vitro*, but this recruitment does not occur when the ARF-binding site is mutated (26). In addition, ARF-binding GGA mutants are completely cytosolic when expressed in mammalian cells (27,21).

While carrying out immunofluorescence labeling on cells expressing a CD8-CIMPR chimera, we were struck by the observation that the GGA staining looked much brighter than in non-transfected cells, suggesting either that the GGAs were more highly expressed in these cells, and/or that there were more GGAs associated with membranes. We have followed up this observation in several ways: by comparing the GGAs with other coat proteins in CD8-CIMPR-expressing cells, by comparing the CD8-CIMPR chimera with other cargo proteins, by assaying total and membrane-associated GGAs in cells expressing different constructs and by using an *in vitro* recruitment assay to investigate the mechanism involved.

## Results

### Localization of coat proteins in CD8-CIMPR-expressing cells

**Figure 1:**
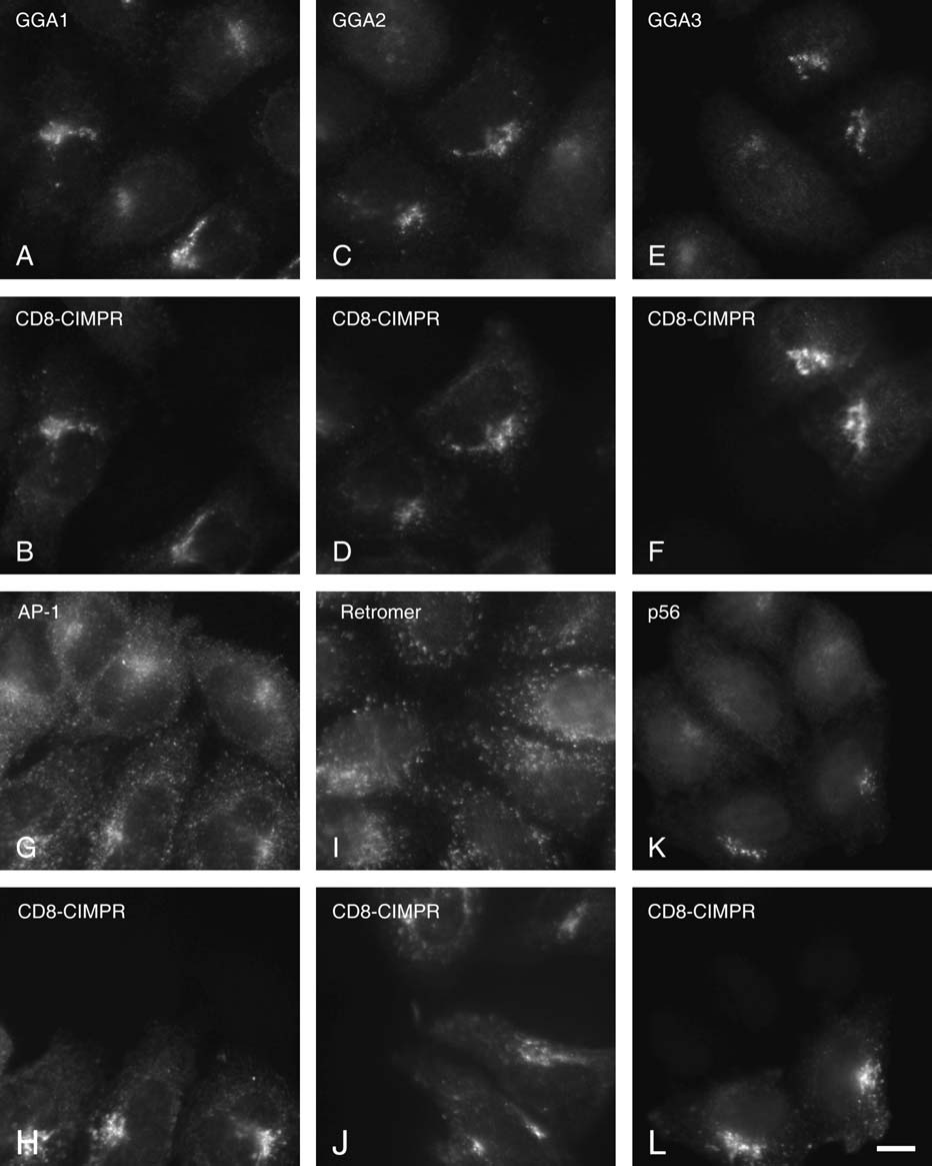
Expression of a CD8-CIMPR chimera enhances GGA labeling Cells stably expressing CD8-CIMPR were mixed with non-transfected cells, fixed and double labeled with anti-CD8 (B, D, F, H, J and L) and antibodies against various coat proteins (A, C, E, G, I and K, shown above the CD8 images). All three GGAs, as well as their binding partner p56, show enhanced labeling in the CD8-CIMPR-expressing cells; retromer shows no increase in labeling, and AP-1 may show a subtle increase. Scale bar: 10 μm.

We first noticed that cargo proteins affect the localization of GGAs when we were carrying out immunofluorescence experiments on stably transfected HeLa cells expressing a CD8 chimera with the cytoplasmic tail of CIMPR. An antibody that we had raised against GGA2 (13), which had never produced a very strong signal by immunofluorescence, gave unusually bright labeling when used on the CD8-CIMPR-expressing cells. Similar results were seen in transiently expressing cells (unpublished observations). To determine whether other GGAs were also affected by the expression of the CIMPR chimera, we mixed together control and CD8-CIMPR-expressing cells, then double labeled for CD8 and GGA1, GGA2 or GGA3. Figure 1A–F shows that the labeling of all three GGAs is enhanced in the CD8-CIMPR-expressing cells.

The CIMPR has a complex trafficking itinerary, cycling between the TGN, different types of endosomes and the plasma membrane, and it contains a number of different sorting signals in its cytoplasmic tail to facilitate interactions not only with GGAs but also with AP complexes and with the retromer complex (28–30). Thus, we also double labeled for AP-1 and for retromer. Figure 1G–J shows that there may be a subtle effect on AP-1, but retromer labeling looks similar in control and CD8-CIMPR-expressing cells. In contrast, double labeling for p56, a GGA-binding partner, showed enhanced labeling in cells expressing the CIMPR chimera (Figure 1K,L), presumably because p56 follows the GGAs onto membranes.

### Requirement for the DXXLL-sorting signal

**Figure 2:**
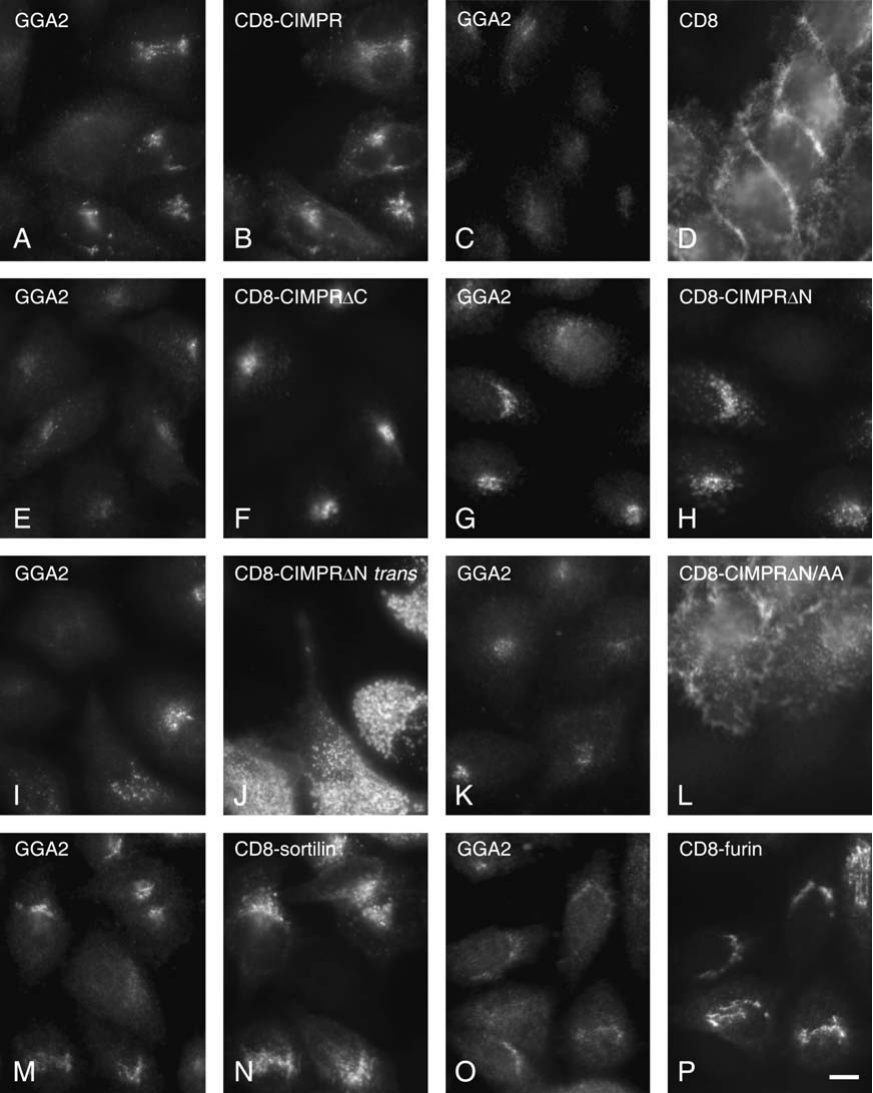
The DXXLL motif is needed for enhanced GGA labeling Cells expressing various CD8 constructs were fixed and double labeled with anti-GGA2 (A, C, E, G, I, K, M and O) and anti-CD8 (B, D, F, H, J, L, N and P). In most of the images, the cells were stably transfected and then mixed with non-transfected cells; however, the cells in (I) and (J) were transiently transfected. Enhanced GGA labeling is only seen in cells expressing constructs with a DXXLL motif in the cytoplasmic tail. Scale bar: 1 μm.

To identify the portion of the CD8-CIMPR chimera responsible for the enhanced GGA labeling, a number of other CD8-based constructs were stably transfected into HeLa cells, and the transfected cells were mixed with control HeLa cells and double labeled for CD8 and GGAs (Figure 2). Although only the results for GGA2 are shown here, by immunofluorescence GGAs 1, 2 and 3 all behaved in the same way (unpublished observations).

Figure 2A–D shows that the enhanced labeling is because of the presence of the CIMPR tail and does not occur in cells expressing CD8 alone. When we truncated the C-terminal end of the CIMPR tail, removing the DXXLL signal (CD8-CIMPRΔC), the construct still had a perinuclear distribution (Figure 2F), but it no longer affected the localization of the GGAs (Figure 2E). In contrast, when we removed the N-terminal portion of the CIMPR tail (CD8-CIMPRΔN), although the construct took on a more peripheral distribution (Figure 2H), GGA labeling was still enhanced (Figure 2G), albeit not as strongly as with the full-length tail. The CD8-CIMPRΔN construct still has a DXXLL motif, but it does not appear to be retrieved as efficiently from endosomes back to the TGN as the construct with the full-length tail, presumably because it lacks other sorting signals. This suggests that there may be less GGA recruitment because there is less of the construct in the appropriate membrane. Consistent with this possibility, transiently transfected cells, which express the CD8-CIMPRΔN construct at much higher levels (Figure 2J), show a more obvious enhancement of GGA labeling (Figure 2I). Mutating the dileucine in CD8-CIMPRΔN to a dialanine causes the construct to go to the plasma membrane (Figure 2L) and abolishes the enhanced GGA labeling (Figure 2K).

We also investigated other CD8 chimeras with tails derived from proteins that cycle between the TGN and endosomes. Figure 2M,N shows cells expressing a construct with its cytoplasmic tail derived from sortilin, which ends with the sequence DEDLLE. It is clear that this construct causes the same enhancement of GGA labeling as some of the CIMPR constructs. In contrast, a CD8-furin chimera, which contains an acidic cluster but no dileucine, fails to enhance GGA labeling (Figure 2O,P). Together, these observations show that the enhanced GGA labeling only occurs when there is a DXXLL-sorting signal in the cytoplasmic tail of the construct.

### GGA2 expression in CD8-CIMPR-expressing cells

**Figure 3:**
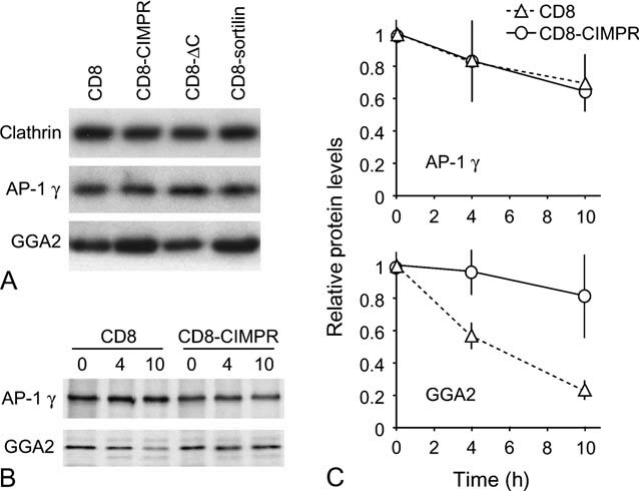
Stability of GGA2 in cells expressing various CD8 constructs A) Equal protein loadings of homogenates of cells stably expressing CD8, CD8-CIMPR, CD8-CIMPRΔC or CD8-sortilin were subjected to SDS–PAGE and Western blots were probed with antibodies against clathrin heavy chain, the AP-1 γ-subunit and GGA2. Quantification of gel bands using a phosphorimager showed a twofold increase in GGA2 in the cells expressing either CD8-CIMPR or CD8-sortilin, both of which have DXXLL motifs. Clathrin and AP-1 expression are unchanged. B) Cells stably expressing either CD8 or CD8-CIMPR were pulse labeled with ^35^S for 15 min, chased for 0, 4 or 10 h, lysed and immunoprecipitated with anti-GGA2 or anti-AP-1. Gel bands were quantified using a phosphorimager and the signal at the 4- and 10-h time-points expressed as a percentage of the signal at the 0 time-point. GGA2 is stabilized approximately fourfold in the CD8-CIMPR-expressing cells compared with the CD8-expressing cells. A graph of the data is shown in (C).

**Figure 4:**
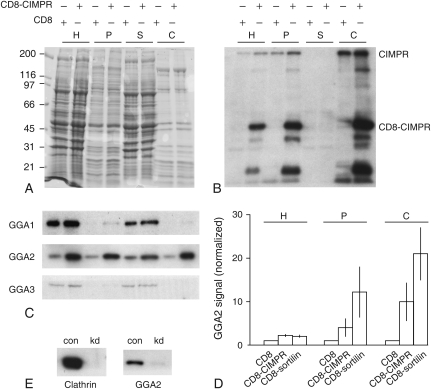
Enrichment of GGA2 in membrane and CCV fractions HeLa cells stably expressing either CD8 or CD8-CIMPR were homogenized and either centrifuged at high speed to produce supernatant and pellet fractions or used to prepare CCV-enriched fractions. Pairs of homogenate (H), pellet (P), supernatant (S) and CCV (C) samples containing equal amounts of protein were subjected to SDS–PAGE and the gels were either stained with Coomassie blue (A) or Western blotted and probed with various antibodies (B and C). B) An antibody against the CIMPR tail, which recognizes both endogenous CIMPR and the CD8 chimera, shows that both proteins are enriched in CCVs and that expression of the chimera does not cause a reduction in the amount of endogenous protein in the CCV preparation. C) Both GGA1 and GGA3 are present mainly in the supernatant fraction, indicating that their association with membranes is labile; however, GGA2 is detectable in both the pellet and the CCV fractions and in both cases the signal is increased in the CD8-CIMPR-expressing cells. D) Blots of the homogenate, pellet and CCV fractions from cells expressing CD8, CD8-CIMPR or CD8-sortilin were quantified using a phosphorimager and expressed as a proportion of control (CD8 expressing) cells. Although GGA2 expression is increased approximately twofold in the CD8-CIMPR- and CD8-sortilin-expressing cells (see also Figure 3), membrane and CCV association are increased still further. E) CCV or mock-CCV preparations were carried out on control (con) and clathrin-depleted (kd) CD8-CIMPR-expressing cells, and equal protein loadings were probed with antibodies against clathrin or GGA2. The loss of GGA2 signal in the preparation from the clathrin-depleted cells shows that the enrichment in the CCV preparation is specific.

Is the increase in GGA labeling in cells expressing constructs with DXXLL-sorting signals caused by an increase in expression levels, increased recruitment onto the membrane or a combination of both? To determine whether GGAs are expressed at higher levels in cells expressing constructs with DXXLL motifs, we analyzed homogenates of cells expressing various constructs by Western blotting with antibodies against GGA2, the AP-1 γ-subunit and clathrin heavy chain (Figure 3A). We found that there is a ∼twofold increase in the level of GGA2 protein in homogenates of CD8-CIMPR-expressing cells (2.19 ± 0.30) and CD8-sortilin-expressing cells (2.03 ± 0.35) compared with cells expressing either CD8 or CD8-CIMPRΔC. This was specific for GGA2 because there were no differences in clathrin (1.03 ± 0.14) or AP-1 (0.93 ± 0.13). Interestingly, we also did not see differences in expression levels of GGA1 or GGA3 (see Figure 4). Hence, the enhancement of GGA1 and GGA3 labeling that we see by immunofluorescence most likely represents the redistribution of cytosolic protein onto the membrane, whereas the enhancement of GGA2 labeling must be due at least in part to its increased expression level.

The increase in expression of GGA2 in cells expressing DXXLL constructs could be caused either by an increased rate of synthesis or by a decreased rate of degradation. To assay the rate of degradation of GGA2, either CD8-expressing cells or CD8-CIMPR-expressing cells were metabolically labeled with ^35^S, chased for either 4 or 10 h, lysed and immunoprecipitated using antibodies against either GGA2 or the AP-1 γ-subunit (Figure 3B). Quantification of the bands using a phosphorimager (Figure 3C) showed that GGA2 was degraded approximately four times more slowly in CD8-CIMPR-expressing cells than in CD8-expressing cells (81 ± 25% of the protein remaining after 10 h compared with 22 ± 5% of the protein remaining). In contrast, similar rates of AP-1 degradation were seen in the two cell lines. Interestingly, GGA1 was found to be remarkably stable in pulse chase experiments, with no significant degradation over the 10-h chase period (unpublished observations). This further supports the idea that GGA2 is regulated differently from GGAs 1 and 3 and, probably, explains why we only see an increase in the total amount of GGA2 in cells expressing DXXLL constructs.

### Association of GGAs with membrane fractions

Our previous studies have shown that the association of GGAs with membrane is highly labile (19), which may account for their absence in preparations of CCVs from rat liver (13). To determine whether the membrane association of GGAs is stabilized in the CD8-CIMPR-expressing cells, we centrifuged homogenates of cells expressing either CD8 or CD8-CIMPR at high speed to separate membranes and cytosol, and we also prepared CCV-enriched fractions from both cell lines. Pairs of samples containing equal amounts of protein were subjected to SDS–PAGE followed by Western blotting (Figure 4). When the blots were labeled with an antibody against the CIMPR tail (Figure 4B), we found that both endogenous CIMPR and CD8-CIMPR are highly enriched in the isolated CCV fraction, with no loss of endogenous CIMPR in CCVs from the chimera-expressing cells, indicating that we have not saturated the sorting machinery for the endogenous protein. Indeed, there is a subtle but consistent increase in the level of endogenous CIMPR in CD8-CIMPR-expressing cells not only in the homogenates but also in the CCVs. However, this increase does not correlate with an increase in the sorting efficiency of cathepsin D (unpublished observations).

The blots were also probed with antibodies against the three GGAs (Figure 4C). Both GGA1 and GGA3 were found to fractionate mainly with the cytosol, consistent with our previous observations, indicating that the membrane association of the GGAs is very labile. Nevertheless, weak labeling for GGA1 could be detected in the membrane pellet and CCV-enriched fraction of CD8-CIMPR-expressing cells, but not of CD8-expressing cells. However, the most striking differences were observed for GGA2. In addition to its twofold increase in the homogenates of CD8-CIMPR-expressing cells (Figures 3A and 4D), GGA2 is enriched approximately fivefold in the high-speed membrane-containing pellet and ∼12-fold in isolated CCVs. Moreover, when we carried out similar fractionation experiments on cells expressing the CD8-sortilin chimera, we found an even greater enrichment of GGA2 in the membrane-containing pellet and CCV fraction (Figure 4D), possibly because the construct is expressed at higher levels. To confirm that the GGA2 is really associated with CCVs and not just cofractionating into the same pellet, we carried out a mock-CCV preparation on clathrin-depleted cells. Figure 4E shows that most of the GGA2 signal disapppears in the clathrin knockdown preparation, confirming that it is a *bona fide* CCV component. Thus, while there is more GGA2 expressed in CD8-CIMPR-expressing cells because of its increased stability, it also redistributes from cytosol onto membranes and is incorporated more efficiently into CCVs. Because by immunofluorescence GGA1 and GGA3 show enhanced perinuclear labeling in CD8-CIMPR-expressing cells, it is likely that they are also enriched in CCVs *in vivo* but are mostly lost from the preparation because of their labile association with membranes.

### GGA recruitment in vitro

**Figure 5:**
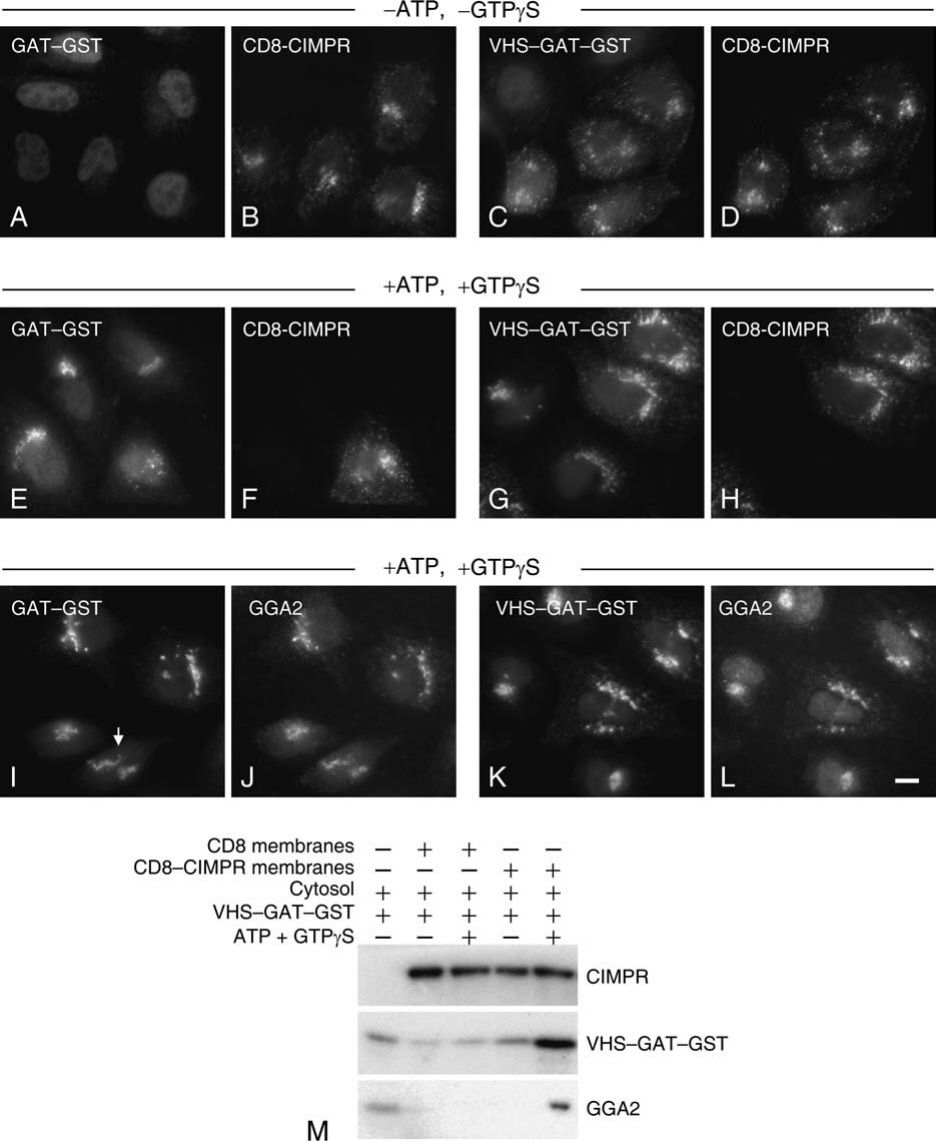
Recruitment of GGA2 constructs onto cell membranes *in vitro* A–H) Cells stably expressing CD8-CIMPR were mixed with non-transfected cells, freeze-thawed and incubated with pig brain cytosol, spiked with either a GAT–GST fusion protein (A, B, E and F) or a VHS–GAT–GST fusion protein (C, D, G and H), either without (A–D) or with (E–H) ATP, an ATP-regenerating system and GTPγS. The cells were then fixed and double labeled with anti-GST (A, C, E and G) and anti-CD8 (B, D, F and H). The VHS–GAT construct is recruited onto membranes of the CD8-CIMPR-expressing cells even in the absence of nucleotides. I–L) CD8-CIMPR-expressing cells were freeze-thawed and incubated with pig brain cytosol spiked with either GAT–GST (I and J) or VHS–GAT–GST (K and L) and ATP, an ATP-regenerating system, and GTPγS. The cells were then fixed and double labeled with anti-GST (I and K) and anti-GGA2 (J and L). The VHS–GAT construct shows better colocalization with GGA2 than the GAT construct [note its reticular appearance (white arrow)]. Scale bar: 10 μm. M) Either CD8- or CD8-CIMPR-expressing cells were permeabilized by freeze-thawing, scraped from the dish and incubated with cytosol spiked with VHS–GAT–GST, either with or without ATP, an ATP-regenerating system and GTPγS. The membranes were pelleted and subjected to SDS–PAGE, Western blotted and probed for endogenous CIMPR, VHS–GAT–GST or GGA2. Both VHS–GAT–GST and GGA2 show enhanced recruitment in the CD8-CIMPR-expressing cells in the presence of nucleotides.

Can we mimic the effect of cargo proteins on GGA recruitment using an *in vitro* system? We and others have previously shown that cytosolic GGAs and recombinant GGA domains expressed as fusion proteins can be recruited onto the membranes of permeabilized cells (13,26), so we compared control and CD8-CIMPR-expressing cells for their ability to recruit GGAs *in vitro*. The two types of cells were mixed together and permeabilized by freeze-thawing, which causes endogenous GGAs to dissociate from membranes (19). We then incubated the cells with pig brain cytosol, which had been spiked with either a GGA2 GAT–glutathione S-transferase (GST) fusion protein or a GGA2 VHS–GAT–GST fusion protein. Immunofluorescence double labeling with anti-GST and anti-CD8 showed that in the absence of nucleotides, there was no detectable recruitment of the GAT domain construct, whether or not the cells were expressing the CD8-CIMPR chimera (Figure 5A,B). However, the VHS–GAT construct, which contains the DXXLL-binding site, was recruited to some extent onto CD8-CIMPR-containing membranes even without ATP or GTPγS (Figure 5C,D). Addition of ATP, an ATP-regenerating system and GTPγS significantly increased the recruitment of the VHS–GAT construct and caused the GAT construct to be recruited as well (Figure 5E–H). The GAT domain construct showed no preferential recruitment in the CD8-CIMPR-expressing cells, and it localized fairly tightly to perinuclear membranes (Figure 5E,F). In contrast, the VHS–GAT construct showed enhanced recruitment in the CD8-CIMPR-expressing cells (Figure 5G,H), with a more peripheral and punctate distribution than the GAT construct and better colocalization with the CIMPR chimera. Interestingly, a construct consisting of just the VHS domain of GGA2 fused to GST was unable to be recruited onto membranes (unpublished observations), demonstrating that the VHS domain alone is not sufficient.

We also investigated the recruitment of cytosolic GGA2 in CD8-CIMR-expressing cells, using an antibody raised against the C-terminal portion of the protein that does not cross-react with either of the fusion proteins. Figure 5I–L, shows some colocalization of both fusion proteins with GGA2, but more complete colocalization for the VHS–GAT construct (note the more reticular appearance of the GAT–GST labeling in the cell at the bottom of panel I).

To investigate the recruitment biochemically, the permeabilized cell membranes were collected by centrifugation and Western blots were probed with antibodies against GST, GGA2 or (endogenous) CIMPR (Figure 5M). There was a tendency for both of the fusion proteins to pellet non-specifically even without any membranes added, so the signal relative to background was too low for us to interpret experiments with the GAT domain construct (unpublished observations). However, the VHS–GAT construct showed enhanced recruitment in the presence of CD8-CIMPR membranes and nucleotides, and we also saw a strong enrichment of GGA2 under these conditions.

### Sensitivity to brefeldin A

**Figure 6:**
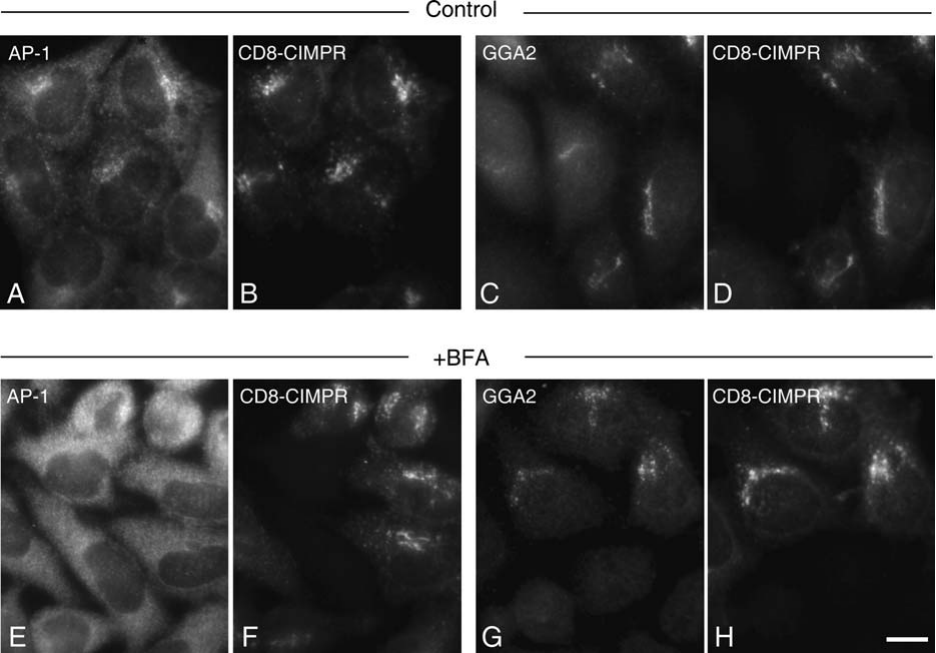
Enhanced GGA2 labeling in CD8-CIMPR-expressing cells is partially BFA insensitive Cells stably expressing CD8-CIMPR were mixed with non-transfected cells, treated with or without 20 μg/mL BFA for 10 min, fixed and double labeled for AP-1 and CD8 (A and B, E and F) or GGA2 and CD8 (C and D, G and H). Addition of BFA causes AP-1 to dissociate completely from the membrane, whereas a pool of GGA2 remains associated in the CD8-CIMPR-expressing cells. Scale bar: 10 μm.

If the VHS–GAT construct is able to be recruited onto membranes of CD8-CIMPR-expressing cells even in the absence of ATP and GTPγS, it may not absolutely need ARF for its membrane localization. Brefeldin A (BFA) is a drug that inhibits ARF by inactivating its nucleotide exchange factors, so we tested the CD8-CIMPR-expressing cells for BFA sensitivity by mixing them with non-transfected cells and treating them with BFA for 2–10 min, then labeling for AP-1, GGA2 and/or CD8. AP-1 dissociated completely from membranes within 2 min of treatment in both non-transfected and CD8-CIMPR-expressing cells (Figure 6E,F, compare with control A and B). However, although GGA2 dissociated from the membranes of non-transfected cells within 2–5 min, in CD8-CIMPR-expressing cells there was still a significant pool of GGA2 associated with membrane even after 10 min (Figure 6G,H, compare with control C and D). This effect was also observed for GGA1 and for the GGA-binding partner p56 (unpublished observations). Thus, increasing the availability of DXXLL motifs not only increases the recruitment of GGA2 onto membranes, it also partially obviates the need for ARF.

## Discussion

Although the GGAs were only discovered in 2000, they have now been extensively characterized both structurally and functionally (reviewed by Bonifacino 25). The structural basis for the binding of all three of the GGA folded domains to various partners has been established. The role of the GGAs in trafficking between the TGN and endosomes has been studied in both yeast and mammalian systems using a combination of genetics, siRNA knockdowns and dominant negative constructs. The recruitment of GGAs onto membranes has also been investigated, but so far all these studies have focused very much on the role of ARF, and the involvement of cargo proteins in GGA recruitment has been essentially unexplored. Here we show that moderate overexpression of cargo proteins with DXXLL motifs significantly increases the association of GGAs with membranes.

The role of cargo proteins in coat protein recruitment in general is still an open question. Several studies have been carried out making use of artificial liposomes with attached peptides containing sorting signals for different coat components, including motifs that bind to AP-1 (31), AP-2 (32) and coatomer (33). All these studies have shown an increase in the binding of coat proteins to liposomes carrying appropriate motifs. However, as discussed in the *Introduction*, the situation is much less clear-cut *in vivo*. For instance, although a recent study showed a ∼40-fold increase in the binding of recombinant AP-2 cores to artificial liposomes containing a YXXΦ peptide when compared with binding to liposomes alone (32), overexpressing constructs with YXXΦ motifs in their cytoplasmic tails has no apparent effect on AP-2 recruitment in HeLa cells [(2,5) and our own unpublished observations]. Similarly, mutating the YXXΦ-binding site on the μ2 subunit of AP-2 decreased binding to YXXΦ-containing liposomes by ∼40-fold (32), but AP-2 complexes containing the same mutation were recruited normally in HeLa and COS cells (34,35). This discrepancy presumably reflects the greater complexity of interactions that take place *in vivo*. AP-2 interacts on the plasma membrane not only with cargo proteins bearing YXXΦ motifs but also with other types of cargo proteins, with phosphatidylinositol bisphosphate (PIP2), and with binding partners for its two appendage domains. In contrast, the liposomes contained only two binding sites for AP-2: YXXΦ motifs and PIP2. What is less clear is whether the inability of cargo to affect the recruitment of AP-2 and other coat proteins *in vivo* is because cargo availability is not a limiting factor or because the cargo is in fact irrelevant.

In the present study, we show that, at least in the case of the GGAs, increasing the availability of cargo proteins causes a striking increase in coat protein recruitment. This is true for all three of the mammalian GGAs, although their regulation appears to be somewhat different. Cells stably transfected with DXXLL-containing constructs were found to express more than twice as much GGA2 as controls, owing to a decrease in the rate of GGA2 degradation. In contrast, expression levels of GGAs 1 and 3 were not affected by cargo. The reason for this differential regulation is not clear, possibly a posttranslational modification is involved [e.g. GGAs 1 and 3, but not GGA2, have been shown to be phosphorylated (36–38)]. But in any case, the increase in GGA2 expression still cannot account for the much higher increase in the amount of GGA2 associated with membranes and with CCVs.

Although our study shows that cargo proteins play a role in GGA recruitment, there are clearly other factors involved. Overexpression of the CD8-CIMPRΔN (DXXLL containing) construct enhances GGA recruitment, but the GGAs show relatively little colocalization with this construct, especially in transiently transfected cells (see Figure 2I,J). This indicates that the cargo must be in the right place in order to contribute to recruitment and that the GGAs are interacting not only with the construct but also with other partners in the same compartment. Similarly, although the VHS–GAT–GST construct was recruited efficiently onto membranes in permeabilized cells, especially if the cells were expressing CD8-CIMPR, a VHS–GST construct was unable to be recruited onto membranes, indicating that the interaction with the DXXLL motif alone is not sufficient for recruitment and that the GAT domain is also involved.

The GAT domain binds to ARF, and there is no question that this interaction is a principal determinant in GGA recruitment (25). However, the ability of the VHS–GAT–GST construct to localize to CD8-CIMPR-positive membranes even in absence of nucleotides and the partial resistance of endogenous GGA2 to BFA in CD8-CIMPR-expressing cells, suggest that ARF is not absolutely essential for recruitment. In addition, it has been shown that yeast GGAs with mutations in the ARF-binding site localize normally (21). Clearly, there are many interactions that contribute to GGA recruitment, fitting in with the notion that docking sites are made up of several components acting together, and that coat proteins – like a number of other biological molecules – are essentially coincidence detectors (39). Thus, in addition to interacting with ARF- and DXXLL-containing cargo to get onto membranes, GGAs are likely to recognize other membrane-associated molecules as well, such as additional types of cargo (e.g. ubiquitinated proteins), phospholipids and/or appendage domain partners.

Perhaps, the most striking effect of overexpressing constructs with DXXLL motifs was the dramatic increase in the amount of GGA2 associated with CCVs, which was about twice as high as the increase in the amount associated with membranes as a whole. This observation suggests that the cargo not only facilitates coat protein recruitment, it may also help to drive the formation of CCVs, as Ehrlich et al. (6) proposed in their live-cell imaging study. One question that we plan to address in the future is whether the cells form more GGA-positive CCVs or whether they form a similar number of CCVs but with more GGAs incorporated into the coat. Either way, our observations show that cargo proteins can make an important contribution to their own sorting, indicating that the coated vesicle is more like an elevator than an escalator.

## Materials and methods

### Plasmid construction

Standard molecular biology techniques were used throughout this study (40). The construction of CD8-CIMPR, CD8-furin and CD8-sortilin in pIRESneo2 has been described elsewhere (30). To generate the CD8-CIMPRΔC (Δ75–163) and CD8-CIMPRΔN (Δ1–75) deletion mutants, the transmembrane and cytoplasmic tail of CD8-CIMPR was first subcloned into pBluescript (Stratagene, La Jolla, CA) using *Eco*RV and *Not*1. CD8-CIMPRΔC was produced by cutting with *Blp*1, digesting with Mung Bean nuclease and then cutting with *Not*1, blunting and religating, resulting in the deletion of amino acids 75–163 of the CIMPR cytoplasmic tail. CD8-CIMPRΔN was produced by cutting CD8-CIMPR in pIRESneo with *Afl*II and blunting with Mung Bean nuclease and then cutting with *Not*1. A *Blp*1 (blunted)-*Not*1 fragment was cloned into the *Afl*II-*Not*1 cut vector, resulting in the deletion of amino acids 1–75 of the CIMPR cytoplasmic tail. The CD8-CIMPRΔN/AA mutant was constructed from CD8-CIMPRΔN using QuikChange mutagenesis (Stratagene) to change the two leucines in the DXXLL motif to alanines. The GGA2 VHS–GAT–GST fusion protein was constructed by amplifying the cDNA encoding residues 1–331 of human GGA2, adding on *Bam*H1 and *Sal*1 cloning sites and then subcloning the polymerase chain reaction product into pGEX4T-1 for GST fusion protein expression. A similar strategy was used to construct the GGA2 GAT–GST fusion protein, amplifying the cDNA encoding residues 157–331. GGA2 VHS–GST was a kind gift from Brett Collins and David Owen (26).

### Antibodies and blotting

Antibodies against GGA3 and CIMPR were kind gifts from Juan Bonifacino (National Institutes of Health, Bethesda, MD, USA) (12) and Paul Luzio (CIMR, Cambridge, UK) (41), respectively. The antibody against CD8 was purchased from Ancell. Rabbit polyclonal antibodies against γ-adaptin (AP-1), clathrin, GGA1, GGA2, p56, GST and mVPS26 were raised in house and have already been described (42,^43^,^13^,^23^,30). The mouse monoclonal antibody against GGA2, used for the *in vitro* recruitment experiments, was a kind gift from Doug Brooks (Women’s and Children’s Hospital, North Adelaide, Australia). Western blots were probed with various antibodies, followed by rabbit anti-mouse (Dako, Glostrup, Denmark) where appropriate and then by ^125^I-protein A as previously described (13). The signal was quantified using a Packard Cyclone phosphorimager.

### Transfection, recruitment and immunolocalization

Constructs were transfected into HeLa using Fugene 6 (Roche, Basel, Switzerland), colonies were selected for stable expression, and clonal cell lines were isolated. For immunofluorescence, stably expressing cells were mixed with non-transfected cells and were fixed either with 3% paraformaldehyde, followed by permeabilization with 0.1% Triton-X-100, or with methanol/acetone (42). For some experiments, the cells were treated with 20 μg/mL BFA for up to 10 min before fixation. Recruitment experiments were carried out on permeabilized cells using pig brain cytosol (42), supplemented with 50 μg/mL recombinant GGA2 GAT–GST or GGA2 VHS–GAT–GST [prepared as described by Page and Robinson (43)], either in the presence or absence of GTPγS, ATP and an ATP-regenerating system, as described by Seaman et al. (42). Primary antibodies are described above; secondary antibodies were purchased from Molecular Probes (Invitrogen, Paisley, UK). Cells were viewed using a Zeiss Axiophot fluorescence microscope equipped with a CCD camera (Princeton Instruments, Princeton, NJ) and photographs were recorded using ip labs software.

### Clathrin-coated vesicle isolation and RNAi interference

The isolation of CCVs from HeLa cells has been described elsewhere (44). Briefly, eight 9-cm diameter tissue culture dishes of HeLa cells stably expressing CD8 chimeras were rinsed and scraped into ice-cold buffer A (0.1 m 2-morpholinoethanesulfonic acid, pH 6.5, 0.2 mm EGTA, 0.5 mm MgCl_2_, 0.02% NaN_3_, 0.2 mm phenylmethylsulphonyl fluoride), homogenized using a motorized Potter glass homogenizer and centrifuged in a Beckman S4180 rotor (Fullerton, CA, USA) at 4800 ×***g*** for 32 min. The postnuclear supernatants were treated with RNase A and the membranes pelleted by spinning at 50 000 ×***g*** for 30 min in a Beckman TLA100.4 rotor. The resulting pellet was resuspended in buffer A, mixed with an equal volume of 12.5% Ficoll/12.5% sucrose and centrifuged in a TLA100.4 rotor at 20 000 ×***g*** for 25 min. The supernatant was diluted with four volumes of buffer A, the CCVs recovered by spinning in a TLA100.4 rotor at 50 000 ×***g*** for 30 min, and the resulting pellets resuspended in buffer A. Yield was determined by quantifying the volume and protein concentration at each step, and then probing Western blots of equal protein loadings with antibodies followed by ^125^I-protein A and quantifying the bound radioactivity using a phosphorimager.

Clathrin-coated vesicles were also prepared from cells depleted for clathrin using RNAi (45). Briefly, HeLa cells stably expressing CD8-CIMPR were transfected with the chc-2 duplex using Oligofectamine (Invitrogen, Carlsbad, CA, USA), as specified by the manufacturer. For efficient knockdown, two transfections were performed 2 days apart, and experiments were carried out 2 days after the second knockdown.
